# Insights into the Mechanisms That May Clarify Obesity as a Risk Factor for Multiple Sclerosis

**DOI:** 10.1007/s11910-018-0827-5

**Published:** 2018-03-10

**Authors:** Marije J. D. Huitema, Geert J. Schenk

**Affiliations:** 1 Department of Anatomy and Neurosciences, Neuroscience Amsterdam, VUmc MS Center Amsterdam, VU University Medical Center, De Boelelaan 1108, 1081 HV Amsterdam, Netherlands; 2 Faculty of medicine, VU University, Van der Boechorststraat 7, 1081 BT Amsterdam, Netherlands

**Keywords:** Obesity, Body mass index, Leptin, Gut microbiota, Multiple sclerosis, Immune system

## Abstract

**Purpose of Review:**

The proportion to which genetic and environmental factors contribute to the etiology of multiple sclerosis (MS) is still incompletely understood. An interesting association between MS etiology and obesity has recently been shown although the mechanisms underlying this association are still unknown. We propose deregulated gut microbiota and increased leptin levels as possible mechanisms underlying MS etiology in obese individuals.

**Recent Findings:**

Alterations in the human gut microbiota and leptin levels have recently been established as immune modulators in both MS patients and obese individuals. A resemblance between pro-inflammatory bacterial profiles in MS and obese individuals was observed. Furthermore, elevated leptin levels push the immune system towards a more pro-inflammatory state and inhibit the regulatory immune response.

**Summary:**

Deregulated gut microbiota and elevated leptin levels may explain the increased risk of developing MS in obese individuals. Further research to confirm causality is warranted.

## Introduction

Multiple sclerosis (MS) is a chronic disease, characterized by demyelination and neurodegeneration in the central nervous system (CNS), resulting in neurological deficits and a wide range of functional disabilities [1]. Worldwide, MS affects approximately 2.3 million people, generally starting in early adulthood. The most common form of MS is relapsing-remitting MS (RRMS), which is characterized by multiple lesions in the brain and spinal cord, typically associated with a clinical event and subsequently complete or partial recovery [2, 3]. Although the cause of MS is still largely unknown, both genetic and environmental factors are involved. To date, the leukocyte antigen HLA-DRB1*15 allele is the most common genetic marker for MS susceptibility [4]. However, individuals carrying this allele may have only a slightly increased risk of developing clinically definite MS [5]. This suggests that additional factors influence disease onset. An interesting environmental factor that has recently been associated with MS etiology is obesity [6]. Due to dietary changes, especially in the western world, obesity has become epidemic. Approximately 35% of individuals meet criteria for overweight (body mass index (BMI) 25–30 kg/m^2^) or obesity (BMI > 30 kg/m^2^) worldwide. Moreover, obesity creates a chronic state of low-grade inflammation, which is possibly associated with an increased risk of developing MS [7–9]. The mechanism responsible for this increased risk of developing MS is still unknown [10]. Two possible hypotheses are discussed in this review.

The first hypothesis suggests that gut microbiota may play a crucial role. The human gut microbiota composition is associated with other metabolic and autoimmune diseases [11]. The gut microbiota consists of the entire genome of microbes in the gut, including bacteria, viruses, and fungi [4]. It plays an essential part in digestion optimization, immune system development, and protection against pathogenic microorganisms [4]. Interestingly, changes in the microbe composition are able to regulate CNS function and vice versa. Several specific bacteria have been associated with immune and inflammatory functions in MS patients and might therefore be an important factor for developing MS in genetically predisposed individuals. In addition, alterations in the microbiome are associated with body-weight [10]. If the gut microbiota compositions of MS patients and obese individuals carry significant similarities, and both have differences from non-obese persons, this might explain mechanisms for MS susceptibility in obese individuals.

The second hypothesis involves leptin levels. Leptin is a peptide hormone produced by adipocytes. Its main function is regulating energy expenditure, by monitoring the fat storage and determining food intake via signaling satiety [12, 13]. Recently, leptin was found to have powerful pro-inflammatory effects [14]. Moreover, leptin concentrations are positively correlated with BMI, and a link between leptin and MS disease progression has been suggested [12, 15]. Thus, increased leptin levels may support the association between MS etiology and obesity.

### Obesity as a Risk Factor for MS Onset and Its Impact on Disease Progression

The association between obesity and MS has been investigated extensively. According to a retrospective case-control study, which included 1571 MS cases and 3371 controls, the average weight among MS patients was higher compared with that of their non-MS control subjects during non-diseased adolescence. Furthermore, a BMI above 27 kg/m^2^ at age 20 resulted in a twofold increased risk of developing MS. Subjects with a BMI ranging between 25 and 27 kg/m^2^ at age 20 also had a slightly increased risk. This association was not found between their current adult BMI and risk of MS [16]. Langer-Gould (2013) stated that an increased risk of developing pediatric MS or clinically isolated syndrome was only observed for obese girls and not for boys, even though obesity was more prevalent in boys [17].

A prospective, longitudinal study reported a similar twofold increase in MS risk when obesity was present during childhood. In addition, pre-puberty overweight and pubertal overweight yielded the same risk of MS. This association was significantly stronger in girls [18].

Moreover, a genetically determined elevated BMI is associated with a 41% increased risk of MS [19•]. While obesity in early life may be a risk factor for MS development, it might also relate to an earlier onset of symptoms. According to Kavak et al. (2015), women with overweight and obesity at age 25 had a significantly earlier onset of the disease [20].

Interestingly, in normal-weight subjects, the HLA-DRB1*15 allele contributed to a 2.9-fold increased risk for developing MS. The presence of both obesity and the HLA-DRB1*15 allele led to a 9.1-fold increase in MS risk compared with normal-weight, non-carriers of this allele. These findings strongly support the notion that being obese is an environmental risk factor for developing MS, especially in genetically predisposed individuals [20, 21•]. Interestingly, several studies indicate that despite being a risk factor for acquiring MS, obesity itself does not affect progression of the disease [20, 22–24].

### Possible Mechanisms for the Increased Risk of MS Susceptibility in Obese Patients

#### The Gut Microbiota

Animal studies using experimental autoimmune encephalomyelitis (EAE) mice as an MS model show that antibiotics which reduce the gut commensal bacteria impair the development of EAE and reduce EAE severity. This was mainly associated with a reduction of pro-inflammatory cytokines in the circulation and an increase in regulatory T cells (T_reg_), which have anti-inflammatory properties. In addition, germ-free mice seem to be resistant to developing EAE or had a milder disease course [25]. In contrast, when mice were colonized with segmented filamentous bacteria, they developed EAE with a more severe disease course. In this case, the CNS contained increased pro-inflammatory Th1 and Th17 cells, suggesting that the gut microbiota are able to mediate immune responses in other tissues [10, 25, 26].

##### The Gut Microbiota in MS Patients

A number of studies have investigated the microbiome in MS patients. Results are summarized in Table 1. Jangi et al. (2016) examined stool samples of 60 RRMS patients and 43 healthy controls (HCs). The results showed significantly increased *Methanobrevibacter* (phylum *Euryarchaeota*) and *Akkermansia* (phylum *Verrucomicrobia*) genera and decreased *Butyricimonas* (phylum *Bacteroidetes*) levels in the feces of patients treated with beta-interferon/glatiramer acetate and untreated MS patients (no treatment for a month) compared with HCs [38•]. From this cohort, patients and HCs with the highest and lowest *Methanobrevibacter* populations were selected, which resulted in 18 MS patients and 18 HCs. Five hundred sixty-eight immune-related genes from peripheral blood CD4^+^ T cells and CD14^+^ monocytes were studied. In MS patients, both *Methanobrevibacter* and *Akkermansia* were positively correlated with a set of genes expressed in T cells, namely CASP1, TRAF5, and STAT5B [38•]. These genes are involved in interferon (IFN) signaling, interleukin (IL)-2 signaling, and PPAR and RXR pathway activation. The TRAF5 pathway regulates T cell activation and is known to be overexpressed in MS, as is STAT5B [45]. *Butyricimonas* was negatively correlated with these genes in T cells, and in HCs, the correlation was nearly zero. Moreover, *Butyricimonas* positively correlated with the anti-inflammatory cytokine TNFA1P3 and with NFKBIA, which are downregulated in MS [45, 46].

**Table 1:** Summary of the phylum and species of the gut microbiota that are altered in MS patients and obese patients in comparison to healthy controls, showing if species are reduced, increased, or unchanged in the gut microbiota

Phylum	Bacteria	Immune function	Multiple sclerosis	Obesity
Bacteroidetes	*Butyricimonas*	Anti-inflammatory (butyrate-producing) [1]	***↓***[25], ***↓***[27], ***↓***[28]*	
Obesity: ***↓***	*Prevotella*		***↓***[25], ***↓***[29], ***↓***[30], **↑**[28]*	**↑**[31], ↔[32••], ↔[33]
[34, 35]	*Bacteroides*		***↓***[29]	***↓***[32••], ***↓***[28]
MS: ***↓*** [30]	*Parabacteroides*		***↓***[30]	
	*Flavobacterium*		**↑**[30]	
	*Pedobacter*		**↑**[30]	
*Euryarchaeota*	*Methanobrevibacter (smithii)*	Pro-inflammatory [1]	**↑**[25], **↑**[27], **↑**[28]*	**↑**[36], **↑**[31], ↔[37]*, ***↓***[35]
*Verrucomicrobia*	*Akkermansia*	Pro-inflammatory [1]	**↑**[25], **↑**[27], **↑**[28]*	***↓***[31], ***↓***[27]
*Firmicutes*	*Faecalibacterium Prausnitzii*	Anti-inflammatory (butyrate-producing) [2]	***↓***[29] ↔[30]	**↑**[33]
Obesity: **↑** [32••]	*Clostridia cluster XIVa and IV*	(Anti-inflammatory: some are butyrate-producing) [5]	***↓***[29]	
	*Clostridium perfringens* (*cluster I)*		***–***	↔[32••]
	*Streptococcus*		**↑**[29]	
	*Lactobacillus*		***↓***[30], **↑**[28]*	↔[32••] ↔[33], **↑**[37]*, **↑**[35]
	*Erysipelotrichaceae*	(Anti-inflammatory: some are butyrate-producing) [5]	***↓***[30]	**↑**[31]
	*Blautia*		**↑**[30], **↑**[28]*	
	*Dorea*		**↑**[30], ***↓***[28]*	**↑**[28]
	*Anaerostipes*		***↓***[29]	
	*Lachnospiraceae*		***↓***[25], ***↓***[30], ***↓***[28]*	**↑**[28]
*Actinobacteria*	*Collinsella*		***↓***[25], ***↓***[30]	
	*Slackia*		***↓***[25]	
	*Bifidobacterium*		**↑**[29], **↑**[28]*	↔[32••], ↔[33], ***↓***[37]*
	*Adlercreutzia*		***↓***[30]	
*Proteobacteria*	*Pseudomonas*		**↑**[30]	
	*Mycoplana*		**↑**[30]	
	*Sutterella*		***↓***[29], **↑**- therapy [25]	
	*Haemophilus*		***↓***[30]	

References: [38•]. Jangi et al. (2016), [39]**.** Miyake et al. (2015), [40••]**.** Chen et al. (2016), [32••]**.** Mbakwa et al. (2015), [33]**.** Zhang et al. (2009), [34]. Zuo et al. (2011) [41]**.** Balamurugan et al. (2010) [42]**.** Ley (2006). [27]. Cantarel et al. (2015) [28]. Tremlett (2016), [37]. Yun et al. (2017), [35]. Liu et al. (2017), [43]. Ignacio et al. (2015), [44]. Million et al. (2013)

*Pediatric

In monocytes of MS patients, *Methanobrevibacter* and *Akkermansia* were also positively correlated with several genes, namely MAPK14, MAPK1, LTBR, STAT5B, CASP1, and HLA-DRB1. Whereas *Butyricimonas* had negative correlations with these genes. They are involved in the maturation of dendritic cells (DCs) and INF signaling. MAPK genes in monocytes can activate the immune system and play a role in MS pathogenesis [45]. In untreated MS patients, *Akkermansia* was positively correlated, and *Butyricimonas* was negatively correlated with these genes in monocytes, while these correlations were not found in HCs. This suggests that increased levels of *Methanobrevibacter* and *Akkermansia* might have proinflammatory properties in MS patients, by activating DCs and recruitment of inflammatory cells [47, 48]. A reduced *Butyricimonas* level was also associated with pro-inflammatory gene expression and with less butyrate production [38]. Butyrate normally activates colonic T_reg_ cells, inhibits the inflammatory IL-12, and activates the anti-inflammatory IL-10 in monocytes, which promotes an anti-inflammatory state. Thus, less butyrate production may disrupt gut barrier function and induce inflammation [29, 49].

Miyake et al. (2015) followed a cohort of Japanese HCs and RRMS patients during remission. The phyla *Firmicutes*, *Bacteroidetes*, *Proteobacteria*, and *Actinobacteria* were the major phyla present for both MS patients and HCs. However, the *Bifidobacterium* and *Streptococcus* genera were more abundant, and 19 species were less abundant in MS patients, with 14 of them belonging to *Clostridia* clusters IV and XIV (phylum *Firmicutes*). These *Clostridia* species contain butyrate-producing bacteria [30, 39]. Additionally, several *Bacteroides* (phylum *Bacteroidetes*), *Faecalibacterium prausnitzii*, and *Anaerostipes* were less abundant in MS patients. *Faecalibaterium prausnitzii* belongs also to the butyrate-producing species [50]. Consistent with Jangi et al. (2016), a decrease in *Prevotella* (phylum *Bacteroidetes*) was observed, which is negatively correlated with MS pathogenesis [38•].

Finally, Chen et al. (2016) compared RRMS patients during remission and active phases to HCs. While the overall species richness did not significantly differ between MS and HCs, there was a significant difference between active MS patients and MS patients in remission. Importantly, the gut microbiota composition of patients in remission resembled that of the HCs [40••]. An abundance of the genus *Blautia* (phylum *Firmicutes*) in MS patients and an abundance of *Parabacteroides* (phylum *Bacteroidetes*) in HCs were observed, which implies that MS patients have a gut microbial dysbiosis. Additionally, a prediction model was used, which was consistent with the microbes found with 16S rRNA gene sequencing. Findings are summarized in Table 1 [40••]. Notably, *Prevotella* is reduced and the sub-type *Prevotella histicola* is known to suppress EAE [51]. *Erysipelotrichaceae* is also decreased and has anti-inflammatory effects, by playing a major role in the bile gut metabolism [31, 36]. This suggests that decreased levels of these species play a role in disease exacerbation [40••].

In summary, depletion or enrichment of specific species in the gut microbiota establishes either pro-inflammatory or anti-inflammatory effects and may thus have substantial impact on MS pathogenesis.

##### The Gut Microbiota in Obese Patients

To evaluate a possible parallel with MS patients, the microbiota of obese individuals is evaluated below. Findings are summarized in Table 1.

Mbakwa et al. (2015) examined 472 children and quantified *Methanobrevibacter smithii* and *Methanobrevibacter stadtmanae*, because these were previously associated with overweight. *M. smithii* was present in 369 and *M. stadtmanae* in 39 children. *M. stadtmanae* was not significantly associated with overweight. However, when adjusting for confounders, children with a high count of *M. smithii* were three times more likely to be overweight [32••].

This is in line with Zhang et al. (2009), who observe increased methanogenic *Archaea*, including *M. smithii*, in obese individuals. In addition, *Prevotellaceae* was significantly increased, and *Akkermansia* was reduced in obese individuals compared with normal-weight subjects [33].

Other studies have found conflicting correlations. In a Chinese study of obese subjects (*N* = 52), the level of *Bacteroides* and *Clostridum perfringens* (*C. perfringens*) was significantly lower than in normal-weight subjects (*N* = 52) [34]. A study done by Balamurugan et al. (2010) observed no differences in amount of *Lactobacillus* and *Bifidobacterium* in obese and normal-weight Indian children. Moreover, no significant difference in *Prevotella* amount was observed [41].

Also, a reduced amount of the phylum *Bacteroidetes* and an increased amount of the phylum *Firmicutes* were observed in obese subjects, which are normally beneficial bacteria dominating in the human gut. When obese patients were assigned to a low-calorie diet, the proportion *Bacteroidetes* increased simultaneously with weight loss. This implies that the phylum *Bacteroidetes* is negatively correlated with weight [42].

Le Chatelier et al. (2013) divided individuals into low-gene count (LGC) and high-gene count (HGC) groups. The LGC group represented 23% of the study population and contained a significantly higher level of obese individuals. In addition, LGC obese individuals had lower microbial diversity compared with the HCG individuals. This was associated with a decrease in eight butyrate-producing species in the gut microbiota of LGC individuals. *M. smithii* and *Faecalibacterium* were also decreased. Low bacterial genetic diversity was associated with an increased serum leptin, decreased serum adiponectin, more insulin resistance, and a more marked inflammatory phenotype. This low-grade inflammation and insulin resistance could be due to the reduced amount of butyrate-producing species, which might shift the immune system towards a more inflammatory state.

#### The Role of Leptin in the Immune System

The mechanism that may explain the association between MS pathogenesis and obesity is the presence of significantly increased levels of the adipose tissue hormone leptin in both patient populations [21, 52, 53].

Leptin plays a role in innate and adaptive immunity. The adipokines (leptin, adiponectin, and pro-inflammatory cytokines) are secreted in the circulation and connect the adipose and lymphoid tissue compartments. Adipose tissue differentiation and leptin secretion can be induced by the pro-inflammatory cytokines, IL-1, and tumor-necrosis factor (TNF). Leptin release supports the differentiation of pro-inflammatory T helper 1 (T_H_1) cells. In the presence of leptin, a higher proliferation rate and production of IL-2 is mediated in naïve T cells. However, minimal proliferation of memory T cells is induced, which results in increased production of interferon (INF)-γ. In turn, INF-γ is able to induce T_H_1 responses. Moreover, leptin can act as an early acute phase reactant, similar to C-reactive protein (CRP), IL-6, and IL-1, which are released in high amounts during bacterial infection, sepsis, and inflammation [13].

**Fig. 1:**
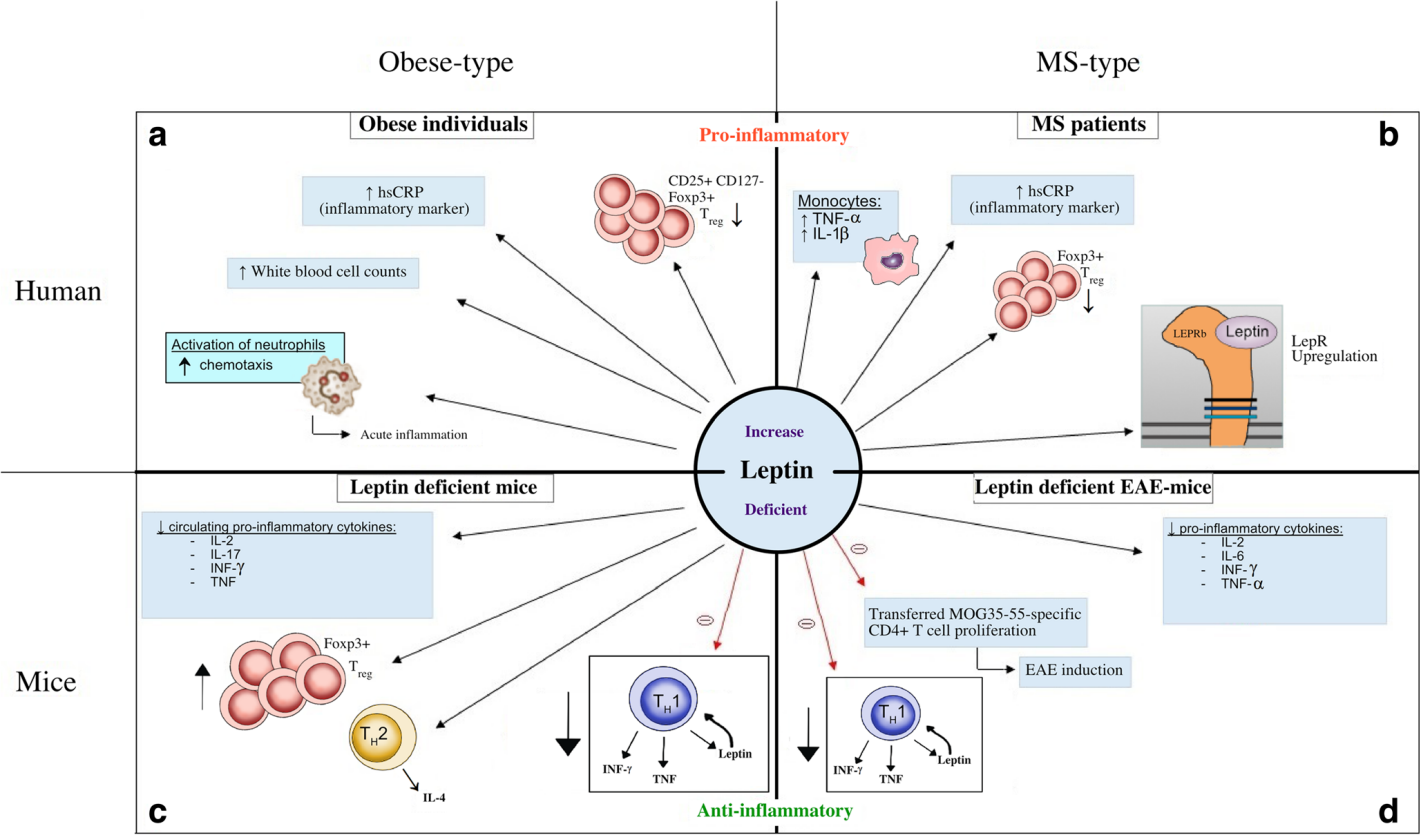
Summary of the effects of leptin in human and mice. Leptin affects the immune system in both mice and human. **a** In MS patients, an increase in leptin causes an upregulation of pro-inflammatory cytokines, a decrease in the anti-inflammatory Foxp3+ T_reg_ cells, and an upregulation of the LepR. **b** Similarly, in obese individuals, a decrease in Foxp3+ T_reg_ cells exists. Moreover, they show an increase in white blood cells and activated neutrophils, which puts the immune system more towards a pro-inflammatory state and induces acute inflammation. **c** In leptin-deficient mice, a decrease in pro-inflammatory cytokines and an increase in Foxp3+ T_reg_ cells are present. **d** Moreover, EAE (induced by MOG35-55-specific CD4+ T cells) is not able to develop when leptin is not present

Leptin acts on the leptin receptor (LEPR) and is expressed by CD4^+^, CD8^+^ human T cells, T_reg_ cells, B cells, monocytes/macrophages, endothelial cells, and natural killer (NK) cells. [13]. LEPR belongs to the class I cytokine receptor family and is therefore able to activate the signaling proteins JAK (Janus Kinase) and STAT (signal transducer and activator of transcription). Subsequently, JAK2 tyrosine kinase will be activated, which in turn activates STAT3. Also, the phosphorylation of STAT3 in CD4^+^, CD25^−^ effector T cells is mediated by leptin. On the contrary, there are no associations with induction of STAT3 phosphorylation in CD4^+^, CD25^+^ T_reg_ cells, which confirms their hypo-responsiveness. T_reg_ cells are a subset of the CD4^+^ T cell that regulate immune suppression and maintain self-tolerance. T_reg_ cells are characterized by promoting Foxp3 (forkhead family transcription factor p3) and increased levels of the IL-2 receptor (CD25) [7, 54]. Neutralizing leptin levels results in a reversal of inactivity and reduced proliferation of T_reg_ cells [12, 55]. This is in line with findings described by Moraes-Vieira et al. (2013), who observed that CD4^+^ T cells of leptin-deficient mice have an increased generating capacity of T_reg_ cells [56]. Figure 1 summarizes the most important effects of leptin.

##### MS and Leptin

Considering leptin as a serious pro-inflammatory modulator, its effects might explain the association between obesity and MS, since leptin is also increased in MS patients [53, 57]. Interestingly, it has been shown that leptin plays a key role in EAE by proliferation of CD4^+^ autoreactive T cells. Leptin-deficient (Lep^ob/ob^) mice injected with transferred encephalitogenic MOG35-55-specific CD4^+^ T cells failed to develop EAE. Moreover, this coincided with reduced pro-inflammatory cytokines (IL-2, IL-6, INF-γ, TNF-α) and a reduced cell proliferation of transferred MOG35-55-specific CD4^+^ T cells. In contrast, the MOG35-55-specific CD4^+^ T cells did induce EAE in Lep^ob/ob^ mice with leptin replacement. Treatment with leptin also restored the reduced proliferation and pro-inflammatory cytokine levels in Lep^ob/ob^ mice [58]. Moreover, R-EAE mice (a model for RRMS) with lowered leptin levels showed a better clinical outcome, less relapses, and a reduced disease progression [15].

In MS patients, Emamgholipour et al. (2013) observed that leptin is negatively correlated with FoxP3 gene expression in PBMCs and is positively correlated with TNF-α, IL-1β, and hsCRP levels [59]. In addition, during the course of MS, leptin concentrations were significantly increased in female patients [60, 61]. These elevated levels can create a positive feedback loop, which leads to MS progression: higher leptin levels cause a reduction of Foxp3 expression and increased production of pro-inflammatory cytokines. These cytokines lower Foxp3 even more, which in turn cause an increase of leptin and pro-inflammatory cytokines [59].

A leptin increase was observed during the acute phase of the disease in both the CSF and serum of untreated RRMS patients compared to the remitting phase, in contrast with the control group. This is possibly secondary to in situ synthesis of leptin in the CNS or increased transport across the blood–brain barrier upon enhanced systemic production. This was positively correlated with increased INF-γ production in the CSF and inversely correlated with the percentage of circulating T_reg_ cells [57].

Elevated concentrations of leptin have been found in MS patients [62], although Rotondi et al. (2013) found similar leptin levels in patients and healthy controls [61].

In RRMS relapsing patients, an upregulation of the phosphorylated STAT3 in CD4^+^, CD8^+^ T cells, and PBMCs was found. This leads towards upregulation of the leptin receptor in the acute phase of the disease and enhances leptin sensitivity in RRMS patients. These data suggest that the LEPR, rather than leptin itself, might play a role in MS pathogenesis in the acute phase of the disease by upregulating the immune response [63]. This is supported by the finding that no difference in leptin concentration was found between the pre-relapse and remission phases in patients naïve to treatment [62].

##### Obesity and Leptin

The association between obesity and a low-grade inflammatory state of the adipose tissue has frequently been investigated [7, 64]. Wagner et al. (2013) compared non-obese with obese subjects. They isolated PBMCs and found a reduced amount of circulating CD25^+^CD127^−^Foxp3^+^ T_reg_ cells in obese individuals, and this correlated with body weight, BMI, and circulating leptin levels. Furthermore, a significant reduction of T_reg_ cells was also found in individuals that had elevated systemic inflammatory markers (hsCRP) or increased HbA1c (a marker for impaired glucose tolerance), which mainly occurred in obesity [7]. This was in accordance with an observational cohort of adolescents. Obese individuals had increased levels of hsCRP and elevated white blood cell counts, which suggests a chronic inflammatory state. Additionally, high circulating neutrophil numbers were observed in obese individuals, indicating acute inflammation [65].

In a study in rodents, Moraes-Vieira et al. (2013) examined the effects of leptin and observed lower circulating pro-inflammatory cytokines (TNF, INF-γ, IL-2, and IL-17) in Lep^ob/ob^ mice compared with controls. Moreover, they observed a shift towards an anti-inflammatory Th_2_ T cell response and an increase in Foxp3 T_reg_ cells, and CD4^+^ T cells showed a reduced alloreactivity [56]. Above all, when administering leptin to Lep^ob/ob^ mice, leptin induces suppression of the anti-inflammatory Th_2_ response and activates the Th_1_ immune response, by increasing the production of pro-inflammatory cytokines (INF-γ and IL-2) [15, 66]. This, together with a reduction in T_reg_ cells, sustains the plausibility of pro-inflammatory effects of leptin in obese individuals.

## Discussion

The aim of this review is to describe the impact of obesity on MS onset and to highlight the potential mechanisms that may underlie MS etiology in obese patients. To this end, we asked if (1) changes in the composition of the gut microbiota or (2) altered leptin levels are possible explanations for the increased risk of developing MS in obese individuals. We hypothesized that the gut microbiome composition of obese individuals resembles that of MS patients and might therefore be an explanation for the increased risk of MS development.

While strong associations are found between early life obesity and an increased risk of developing MS [16–18, 19•, 20, 21], no associations are found between obesity and disease progression [20, 23, 24].

Similarities between some, but not all, gut microbes were observed in MS patients and obese individuals. An overall reduction in the phylum *Bacteroidete*s in obese individuals was found, which resembles the overall observed reduction in this phylum for MS patients [40••, 42, 44]. Specifically the bacteria *Bacteroides* in this phylum were reduced in both MS and obese subjects. Moreover, the presence of *Bacteroides fragilis* is known to coincide with disease attenuation in EAE. Its capsular polysaccharide A is associated with reducing disease severity, by mediating elevated numbers of FoxP3+ T_reg_ cells in the cervical lymph nodes [4, 67]. Thus, a reduced number of *Bacteroides* in obese individuals might contribute to MS susceptibility, by pushing the immune system towards a more inflammatory state.

In the phylum *Euryarchaeota*, the bacterium *M. smithii* is increased in the gut microbiota of both MS patients and obese individuals. Due to its pro-inflammatory properties, T cells and DCs are activated, which are both correlated with MS pathogenesis. Therefore, elevated levels of *M. smithii* support the MS/obesity microbiome hypothesis.

In contrast, no consensus was found between the microbes *Akkermansia*, *Faecalibacterium prausnitzii*, *Lactobacillus*, and *Prevotella.* In fact, the amount of *Akkermansia*, which is pro-inflammatory and is positively correlated with MS pathogenesis, was reduced in obese individuals. The same applies for the anti-inflammatory bacteria *Faecalibacterium* *prausnitzii* and *Erysipelotrichaceae*, which are decreased in MS and increased in obesity. In addition, a significant reduction of *Prevotella* is observed in MS patients, which is correlated with MS pathogenesis, whereas both an increased amount and no changes were observed in obese individuals [33, 39, 40••, 41]. This suggests that *Prevotella* is not associated with the increased risk of MS in obese individuals. A possible explanation could be the increased intake of carbohydrates in diets leading to an increase of *Prevotella*. Besides variation in diets, there are more possible confounders that might influence the gut microbiota, for instance red wine and aspartame consumption [68]. However, not all included studies have been corrected for food consumption. The modulation of MS and EAE by dietary interventions was recently reviewed extensively [69]. Interestingly, immunomodulatory therapy was associated with increased amounts of *Prevotella* and *Sutterella* in treated MS patients, compared with untreated MS patients and HCs. This implies that immunomodulatory treatment may establish a more normalized gut microbiota composition, at least for some microbes [38•], further underscoring a causal role for gut microbiota in MS.

Longitudinal study designs in larger patient and control groups are necessary to provide suitable clinical, pathological, and genetic data to further investigate the interplay of obesity and MS. If causality indeed exists between obesity and MS, prevention of obesity during childhood will establish a 15% reduction in the number of new MS cases [6]. This offers significant prospects for lifestyle interventions and the use of selective anti- or probiotics to normalize the gut microbiota composition.

The second hypothesis we considered was that increased levels of the fat-derived hormone, leptin contribute to the development of MS in obese individuals, since leptin levels are increased in both obese people and MS patients [21, 52, 53]. Specifically high amounts of leptin were present in the CSF and sera of MS patients and significantly more in the CSF during relapses [15], which is possibly due to synthesis of leptin in the CNS or an increased transport across the blood-brain barrier. This implicates that leptin plays a key role in the inflammation that is taking place in the brain during the course of MS.

Moreover, leptin is capable of reducing T_reg_ cells, inducing neutrophil activation, and increasing white blood cell counts and hsCRP. This could implicate that obese individuals might be more susceptible to developing MS, because their immune system is more skewed towards a pro-inflammatory state.

Caloric restriction seems to increase survival and life span rates in EAE mice. It ameliorates the disease, reduces inflammation, and reduces leptin levels. However, no suppression of the immune system was found in this case. This might suggest a possible lifestyle intervention option for MS, but hitherto little research has been done in humans [12].

More interestingly, female MS patients showed higher serum leptin levels than males, despite having a similar BMI. This gender effect was present in both healthy controls and MS patients. This might partly explain the fact that women are more likely to develop MS than men, with a ratio of almost 3:1 [63, 70].

## Conclusion

In conclusion, alterations in the gut microbiome of obese individuals show similarities to those of MS patients. Alterations in abundance of the microbes *M. smithii*, *Bacteroides*, and in the phylum *Bacteroidetes* might put the immune system towards a more inflammatory state. The phylum *Bacteroidetes* showed the most consistent results in this regard. Therefore, the hypothesis that changes in the composition of the gut microbiota are a possible explanation for the increased risk of developing MS in obese individuals is confirmed for some, but not all phyla. Alterations in some genera did not correspond between MS patients and obese individuals, and this review only defines a possible association, thus further research to confirm causality is warranted.

In light of the hypothesis that altered leptin levels are an explanation for the increased risk of developing MS in obese individuals, we conclude that leptin is a modulating factor that switches the immune system towards a more pro-inflammatory state, by secretion of pro-inflammatory cytokines, upregulation of the leptin receptor, and downregulation of anti-inflammatory T_reg_ cells. Therefore, leptin and leptin receptor levels might be a plausible factor contributing to the increased risk of MS in obese individuals.

Finally, in individuals with a low bacterial genetic gut diversity, more weight gain and higher amounts of leptin were found [71]. This suggests that not only alterations in the gut microbiome composition but also increased levels of leptin are present in obese individuals, together contributing to MS susceptibility.
